# Lipid and fatty acid dynamics by maternal Pacific bluefin tuna

**DOI:** 10.1371/journal.pone.0222824

**Published:** 2019-09-25

**Authors:** Yuko Hiraoka, Yumi Okochi, Seiji Ohshimo, Tamaki Shimose, Hiroshi Ashida, Takuya Sato, Yasuhiro Ando

**Affiliations:** 1 Bluefin Tuna Resources Department, National Research Institute of Far Seas Fisheries, Japan Fisheries Research and Education Agency, Shizuoka-shi, Shizuoka, Japan; 2 Environmental Management Unit, JAPAN NUS Co. Ltd, Shinjuku-ku, Tokyo, Japan; 3 Fisheries Management and Oceanography Department, Seikai National Fisheries Research Institute, Japan Fisheries Research and Education Agency, Nagasaki-shi, Nagasaki, Japan; 4 Research Center for Subtropical Fisheries, Seikai National Fisheries Research Institute, Japan Fisheries Research and Education Agency, Ishigaki-shi, Okinawa, Japan; 5 Faculty of Fisheries Sciences, Hokkaido University, Hakodate-shi, Hokkaido, Japan; Universidad de Cádiz, Facultad de Ciencias del Mar y Ambientales, SPAIN

## Abstract

Lipid and fatty acid composition of female Pacific bluefin tuna (PBF, *Thunnus orientalis*) reproductive and somatic tissues in southwestern North Pacific and Sea of Japan spawning grounds are compared. Total lipid (TL) levels are higher in liver than white muscle tissues. An increased gonadosomatic index (GSI) during the early spawning season coincided with decreased TL. Levels of triacylglycerols (TAG) in PBF liver tissues from the Nansei Islands and Sea of Japan, and white muscle in fishes from the Sea of Japan, decreased during the spawning season, while TAG in ovary tissues did not. Concurrent reductions in TL and increases in GSI early in the spawning season suggest TAG depletion was caused by allocation from liver and white muscle tissues to oocytes, that the liver is one of the important lipid-storage organs in PBF, and this species mostly reliant on capital deposits as a mixed capital-income breeder. Differences of docosahexaenoic acid (DHA) levels between spawning grounds were lower in ovary than in muscle and liver tissues. However, eicosapentaenoic (EPA) and arachidonic acid (ARA) levels that influence egg development and embryo and larval growth are significantly higher in PBF tissues from the Sea of Japan than Nansei Islands, which coincided with larval quality. These suggest a maternal effect exists, with egg quality influencing offspring survival, and that the reproductive strategy of PBF varies according to local variation at each spawning ground.

## Introduction

Pacific bluefin tuna (PBF, *Thunnus orientalis*), stocks of which are exploited by many countries, is one of the most valuable fisheries species in the world [1]. Recent stock assessments have indicated the current PBF spawning-stock biomass to be near a historic low, and the stock to be overfished [2]. However, despite the low spawning-stock biomass, no previously observed [2] clear decline in PBF recruitment is apparent. This may be due to a weak stock recruit relationship [3] or environmental factors such as sea surface temperature (SST) [4–6]. The growth rate during the first two weeks of PBF hatching is critical for survival to recruitment [7–9]. Accordingly, maternal investment in eggs might influence survival [10]. In this study we evaluate the energy allocations and linkage of lipids and fatty acids among mothers and larval PBF.

Lipids and their constituent fatty acids are the main source of metabolic energy for swimming, growth and reproduction in fishes [11]. In reproduction, lipids play an important role in eggs [12, 13], because all lipid classes, polar and neural, can be used as energy sources during embryogenesis and larval development during an endogenous stage. Due to demands for improved production of cultured fishes, many studies have focused on relationships between egg quality and larval performance, such as survival and growth rate, and starvation resistance [14–18].

The quantity of essential fatty acids (EFA), such as docosahexaenoic (DHA), eicosapentaenoic (EPA), and arachidonic (ARA) acid in eggs is particularly important, for these promote hatching success, and larval development and survival capability. Fuiman and Ojanguren [14] reported the amount of DHA in eggs of red drum (*Sciaenops ocellatus*) correlated with larval escape response, even when larval diet had been enriched with DHA. This suggests that maternal provisioning of eggs had an effect on larval escape responses, and, consequently survival. Recruitment of Barents Sea cod stock (Atlantic cod, *Gadus morhua*) was also limited by stored lipid energy in the liver of adult females [19]. Therefore, both the quantity and quality of maternal lipids and fatty acid reserves can influence fish productivity [19–21] and offspring fitness [14, 22, 23].

**Fig 1:**
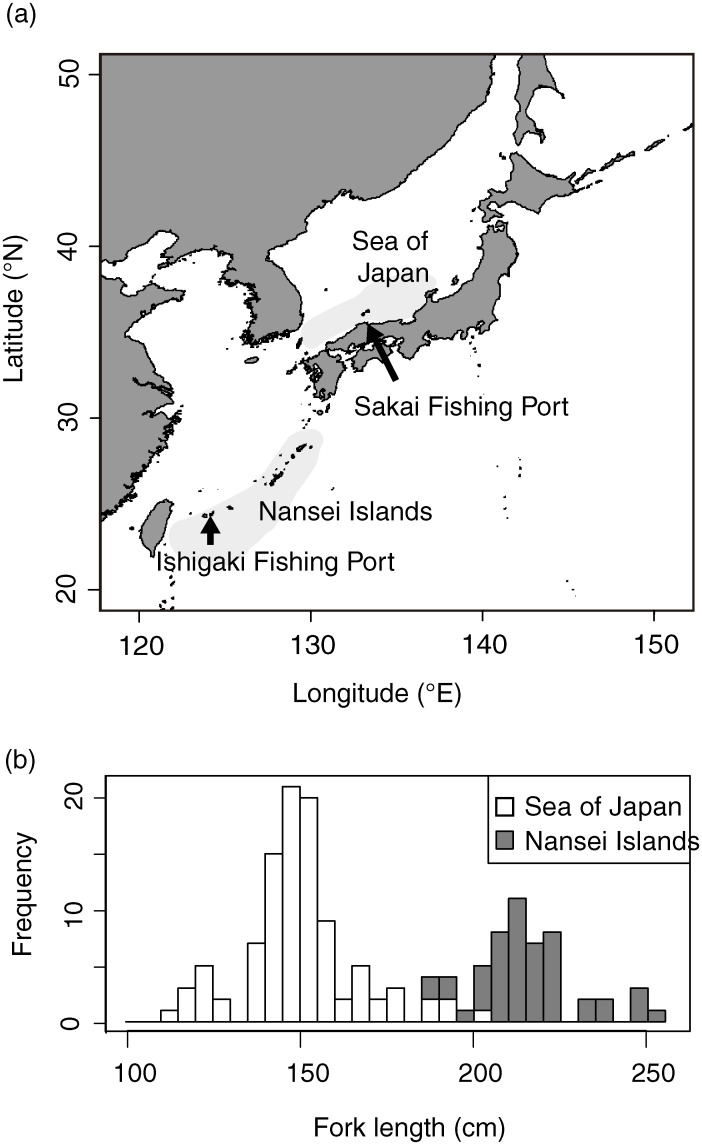
Location of Pacific bluefin tuna major spawning grounds, fishing ports (a), and length composition (b). The digital map data was obtained from Natural Earth.

Based on larval occurrence and ovary histological observations, two major spawning grounds and seasons are recognised for PBF (Fig 1a, [24, 25]). The main spawning ground, from late April to late June, is located northeast of the Philippines and extends to Nansei Islands in the southwestern North Pacific [26–28]. The second is in the Sea of Japan, where spawning occurs from June to August [10, 29]. Most adult female PBF caught during the spawning season in the northwestern Pacific Ocean exceed 10 years age [26], whereas those from the Sea of Japan are predominantly aged 3–6 years [29]. Although the larval survival of PBF depends on their growth in these two spawning grounds, the survival process differs [8, 30–31]. The larval growth until the flexion stage is adversely affected by the lower temperature in the Sea of Japan and has more impact on their survival than around the Nansei Islands [31]. Moreover, PBF larvae caught in the Sea of Japan have more EPA and ARA than those caught around the Nansei Islands [30]. As larval survival is affected by the quantity and nature of lipids and fatty acids, any difference in maternal provisioning of eggs among spawning grounds might affect early life stage survival. How female PBF allocate energy from their tissues to eggs was previously unclear. The aim of this study was to investigate maternal effect on the larval survival process by describing a link between lipids and fatty acids in larval PBF, and the muscle, liver and gonad tissues of female PBF caught during spawning season around the Nansei Islands and in the Sea of Japan spawning grounds.

## Materials and methods

All PBF specimens were caught and handled by commercial fisheries in Japan, and they were not killed for this study. The catch of this species has been controlled based on the assessment of the Western and Central Pacific Fisheries Commission (WCPFC).

### Sample collection

PBF were caught by commercial longline around the Nansei Islands, and purse seine in the Sea of Japan between 2015 and 2017, respectively (Fig 1). At Ishigaki Fishing Port, 51 females from the Nansei Islands were sampled between late April and late June. At Sakai Fishing Port, 100 females from the Sea of Japan were sampled between mid-June and early July. Fishes from each spawning ground were sampled for ovary, muscle, and liver tissues in 2015–2017, 2016–2017, and 2017, respectively (Table 1). Fork length (FL, cm), gilled and gutted body weight (BW, kg), and gonad weight (GW, g) were recorded for each female. A gonad-somatic index (GSI) was calculated for each following GSI = GW×100/BW. White muscle was sampled from near the caudal fin. Ovary, liver and muscle tissues for fatty acid and lipid analyses were frozen. Other ovary sections were fixed in 10% buffered neutral formalin for histological observation.

**Table 1:** Number of samples for each tissue by sampling area and year.

	Nansei Islands			Sea of Japan		
	2015	2016	2017	2015	2016	2017
Ovary	13	14	19	64	13	11
Muscle	0	13	19	0	10	11
Liver	0	0	23	0	0	20

### Histological analysis

Fixed ovary samples were dehydrated in ethanol, embedded in paraffin wax, and thin sections of 5–8 μm were stained with hematoxylin-eosin and slide mounted. Five oocyte stages (perinucleolus (Pn), early yolked (Ey), late yolked (Ly), migratory nucleus (Mn), and hydrated (Hy)) were determined based on the most advanced group of oocytes (MAGO) present, in accordance with previous studies on PBF [10, 26, 29, 32].

### Lipid and Fatty acid analysis

#### Lipid extraction and lipid class analysis

Total lipids were extracted from samples (10 g) following the method of Bligh and Dyer [33]. Lipid content was determined by gravimetry.

Lipid class composition was analysed using thin layer chromatography (TLC) and scanning-densitometry, following Olsen and Henderson [34]. After cleaning in methyl acetate/2-propanol/chloroform/methanol/water (25:25:25:10:9 by volume), total lipids in chloroform (1 mg/mL × 2 μL) were applied to a silica gel 60 plate (10 × 10 cm, 0.25 mm thickness; Merck, Darmstadt, Germany). The plate was developed using the same solvent to a distance of 3.7 cm from the origin. Following drying in a stream of air, the plate was developed by hexane/diethyl ether/acetic acid (80:20:2 by volume) to 7.0 cm. Lipid components were detected by spraying the plate with 10% cupric sulfate in 8% phosphoric acid and by charring at 180°C for 10 min [35]. Lipid class standards (0.5, 1.0, and 1.5 μg) were chromatographed in a similar manner for construction of calibration curves. Chromatograms were scanned using a multifunction printer EW-M571T (Seiko Epson, Suwa, Japan). Densitometry was performed using software ImageJ (http://imagej.nih.gov/ij/).

#### Fatty acid analysis

A portion of the total lipids (10 mg) were converted to fatty acid methyl esters by heating in 7% BF3-methanol (2 mL) at 100°C for 1 h in the presence of toluene (0.5 mL). Methyl esters were purified by column chromatography on silica gel 60 (Merck, Darmstadt, Germany) using a mixture of hexane/diethyl ether (90:10, v/v) for elution.

Methyl esters were analysed using a Shimadzu GC-14A gas chromatograph (Shimadzu, Kyoto, Japan) equipped with a Restek FAMEWAX column (30 m × 0.32 mm i.d., 0.25-μm film thickness; Restek, Bellefonte, PA, USA) and a flame ionization detector. The oven temperature was programmed from 170°C (0 min) to 240°C at a rate of 4°C/min, and held at the final temperature for 24 min. Injector and detector temperatures were 240°C. The carrier gas was helium at a linear velocity of 33.5 cm/s at 170°C (90 kPa). The split ratio was 20:1. Peaks were monitored using a Shimadzu C-R6A integrator.

### Statistical analysis

Two- or three-way ANOVA were applied to evaluate which factors (year, area, or ovarian phase) influenced variation in total lipid content (TL, mg/g) and GSI. To explore seasonal variation in TL, GSI and lipid class composition, we plotted the data from 2015–2017 together, and fitted smoothed curves using locally weighted regressions (LOWESS smoothing) with a standard bandwidth of 0.8.

A Bray-Curtis similarity matrix and permutational multi-variable ANOVA (PERMANOVA) [36, 37] were used to investigate how lipid class and fatty acid profiles varied among tissue sample types and spawning areas. Non-metric multidimensional scaling (NMDS) analysis was performed to visualize differences in lipid class and fatty acid composition. Differences in lipid class and the main fatty acids were examined using similarity of percentages analysis (SIMPER) and Wilcoxon-Mann Whitney tests for each tissue. Statistical analysis used the R software package version 3.3.1 [38], with the “vegan” package used on those data for 59 fatty acids that contributed at least 0.1% to the total fatty acid composition.

## Results

### Length distribution and oocyte developmental stage

**Fig 2:**
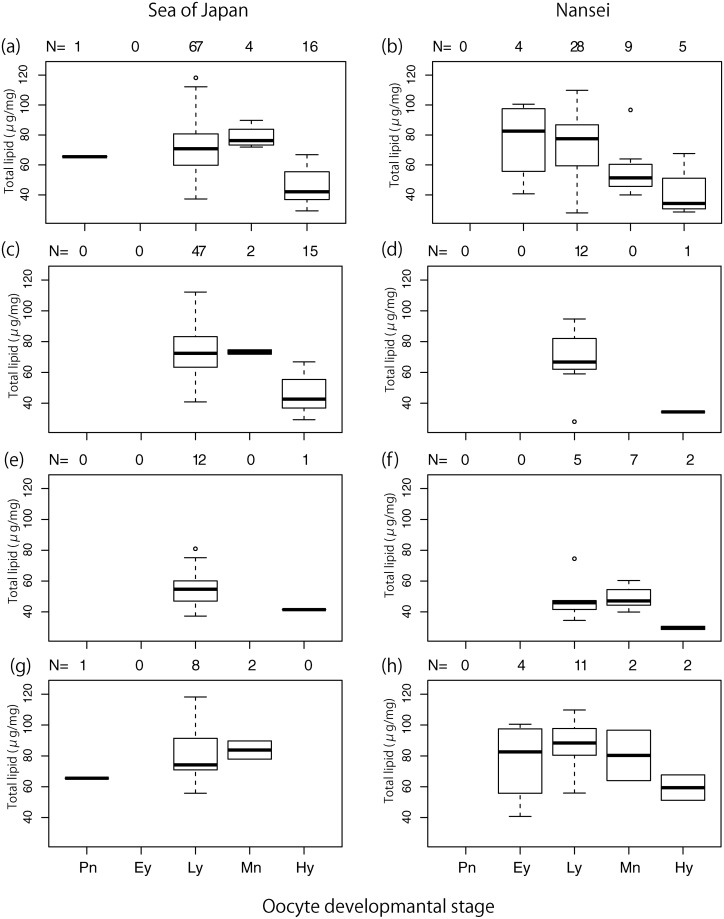
Box plots for total lipids in ovary tissues, separated by oocyte developmental stage: Sea of Japan, all years combined (a), and separately for 2015 (c), 2016 (e) and 2017 (g); and Nansei Island, all years combined (b), and separately for 2015 (d), 2016 (f) and 2017 (h). Oocyte stage: Pn, perinucleous; Ey, early yolked; Ly, late yolked; GVM, germinal vesicle migration; Hy, hydrated.

PBF from around Nansei Island, 187–252 cm FL, were larger than those from the Sea of Japan, 111–204 cm FL (Fig 1b), with peaks at 150 cm (Sea of Japan), and 215 cm (Nansei Islands). Five oocyte developmental stages were observed during the female PBF spawning season: Pn, Ey, Ly, Mn and Hy. In both areas, ovaries identified as Ly were frequent (Sea of Japan: 67, Nansei Islands: 28) compared to other developmental stages (Fig 2a and 2b; less than 16).

### Variation in TL and GSI depending on year, area and oocyte developmental stage

Ovary TL varied significantly with oocyte developmental stage (three-way ANOVA, *P* < 0.001) (Table 2). Significant annual differences in TL were apparent in ovary (three-way ANOVA, *P* < 0.001) and muscle (two-way ANOVA, *P* = 0.010) tissues, but no significant difference in TL was apparent between sampling area for any tissue type. Significant differences in GSI were apparent between oocyte developmental stage and area (three-way ANOVA, *P* < 0.001, *P* = 0.015).

**Table 2:** Anova results (two- and three-way) for tissue-type TL and GSI.

Tissue	Factor	Df	Sum Sq	Mean Sq	F-value	P-value
Ovary	Year	2	13371	6686	30.853	**<0.001**
	Area	1	82	82	0.377	0.54
	Stage	4	11667	2917	13.461	**<0.001**
	Year×Area	2	418	209	0.965	0.384
	Year×Stage	4	224	56	0.259	0.904
	Area×Stage	2	152	76	0.351	0.705
	Year×Area×Stage	1	3	3	0.016	0.901
	Residuals	117	25353	217		
Muscle	Year	1	2738	2737.9	13.424	**<0.001**
	Area	1	5	5.3	0.026	0.873
	Stage	4	762	190.5	0.934	0.454
	Year×Area	1	248	248	1.216	0.277
	Year×Stage	2	614	306.8	1.504	0.234
	Area×Stage	2	254	126.9	0.622	0.542
	Residuals	41	8362	204		
Liver	Area	1	11252	11252	3.111	0.087
	Stage	4	17938	4484	1.24	0.313
	Area×Stage	2	232	116	0.032	0.968
	Residuals	34	122963	3617		
GSI	Year	2	1.84	0.919	0.563	0.5709
	Area	1	9.94	9.938	6.092	**0.015**
	Stage	4	111.64	27.909	17.109	**<0.001**
	Year×Area	2	3.38	1.692	1.038	0.3576
	Year×Stage	4	3.37	0.842	0.516	0.724
	Area×Stage	2	9.94	4.972	3.048	0.0513
	Year×Area×Stage	1	2.77	2.77	1.698	0.1951
	Residuals	117	190.85	1.631		

Although TL of ovary tissues differed significantly between years, similar trends in ovarian stages were apparent between spawning grounds (Fig 2). In any year, TL were relatively high during Ey, Ly and Mn stages, and decreased in the Hy stage (Fig 2c–2h). Considerable variation in TL levels during the Ly oocyte developmental stage was observed, with mean ±SD values of 72.05±14.920 μg/mg, 53.78±13.246 μg/mg and 84.87±16.599 μg/mg, in 2015, 2016 and 2017, respectively.

### Seasonal changes in GSI, TL and lipid class composition

**Fig 3:**
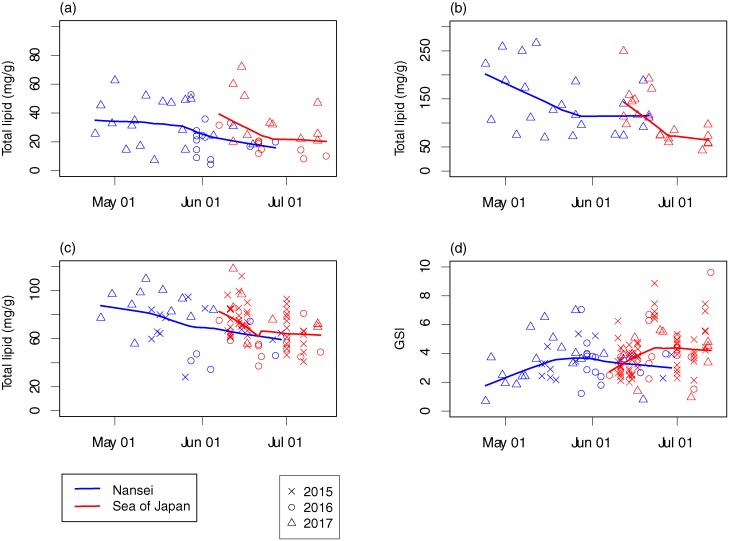
Seasonal change in total lipid in muscle (a), liver (b) tissues, and oocytes (stage Ly) from within the ovary (c), and GSI (d) of female Pacific bluefin tuna around the Nansei Islands (blue) and Sea of Japan (red) in 2015 (crosses), 2016 (open circles), and 2017 (open triangles); with Lowess smoothing curves (bandwidth, 0.8).

Because of significant differences in ovarian TL among oocyte developmental stages, we focused our investigation on temporal variation in the TL of Ly stage developing oocytes. Throughout the spawning period, a decrease in TL was apparent in all tissue samples from females collected in both spawning grounds (Fig 3a–3c). Mean TL values differed by tissue type, being high in liver (126.9 mg/g), intermediate in ovary (71.4 mg/g), and low in muscle (28.5 mg/g) tissues; trend lines in smoothed curves were similar, and were most pronounced in the Sea of Japan, where TL rapidly decreased during June before levelling out to mid-July. Although we did not sample continuously throughout the spawning period each year, TL in muscle and ovary tissues in 2016 was lower than observed in other years (Fig 3a and 3c). In both spawning grounds, trends in GSI were similar, gradually increasing from the onset of the spawning season and then plateauing after mid-spawning season, mid-April and late May off the Nansei Islands, and early and late June in the Sea of Japan, respectively (Fig 3d).

**Fig 4:**
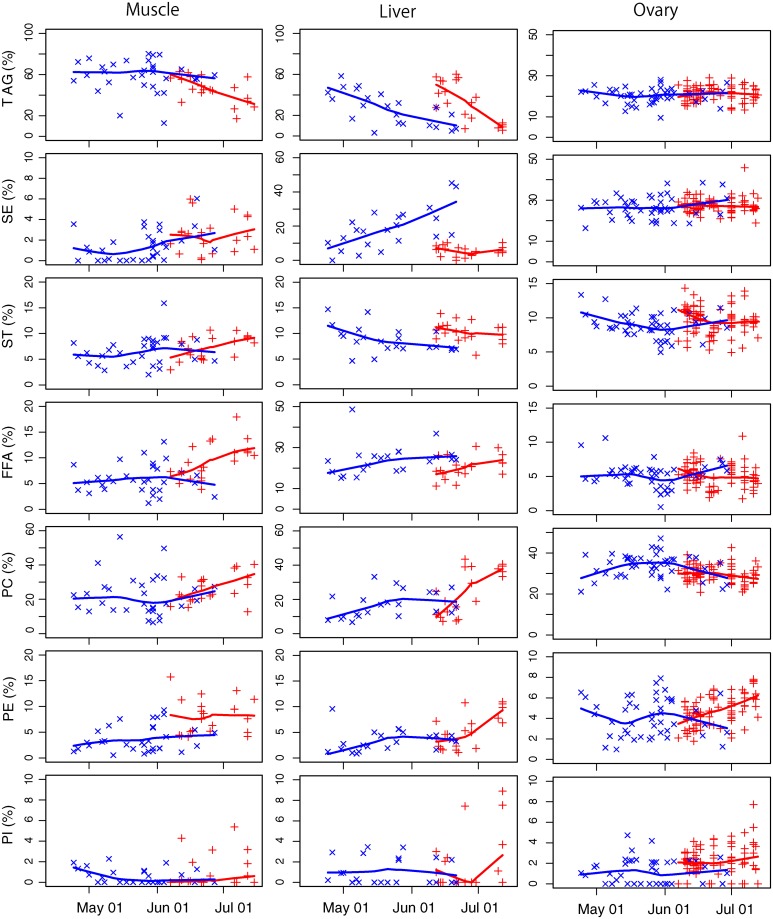
Temporal variation in lipid classes in muscle (left), liver (middle) tissues, and oocytes (stage Ly) from within the ovary (right) of female Pacific bluefin tuna from the Nansei Islands (×) and Sea of Japan (+); with Lowess smoothing curves (bandwidth, 0.8). TAG, triacylglycerols; SE, sterol esters; ST, sterols; FFA, free fatty acids; PC, phosphatidylcholine; PE, phosphatidylethanolamine; PI, phosphatidylinositol.

Temporal changes in seven lipid classes (triacylglycerols (TAG), sterol esters (SE), sterols (ST), free fatty acids (FFA), phosphatidylcholine (PC), phosphatidylethanolamine (PE), and phosphatidylinositol (PI)), are shown in Fig 4. During the spawning season, the main lipid compositions in ovary tissues (TAG, SE and PC) were relatively stable compared to those in muscle and liver tissues (coefficient of variation (CV) 15.5%–17.8% in ovary, 29.7%–88.3% in muscle, and 54.8%–88.8% in liver tissues). The marked depletions in TAG observed in liver tissues were more pronounced in PBF caught in the Sea of Japan than around Nansei Island; TAG in muscle tissues was also depleted in the Sea of Japan from early June to mid-July. Unlike the TAG of liver tissues, TAG in muscle tissues from PBF caught around the Nansei Islands did not decrease, but remained high, albeit highly variable, throughout the spawning season (mean ± SD, 60.5 ± 16.5). Declines in TL and the proportional contribution of TAG in muscle and liver tissues resulted in the smoothed curves of other lipid classes (including SE, PC and PE) trending upwards in both spawning grounds and seasons.

### Differences in lipid and fatty acid composition among tissues and area

**Fig 5:**
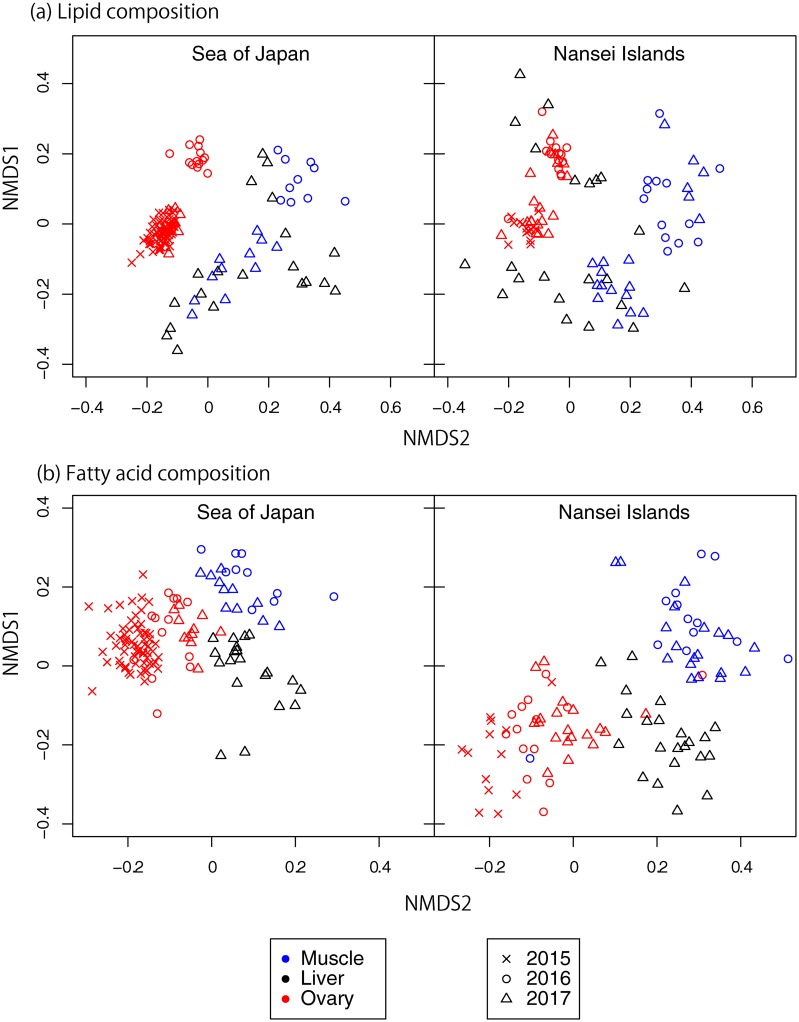
Non-metric multidimensional scaling (NMDS) plot of lipid (a) and fatty acid (b) compositions in ovary, muscle and liver tissues of female Pacific bluefin tuna caught around the Nansei Islands and in the Sea of Japan (two-dimensional stress = 0.16 and 0.17 for lipid and fatty acid compositions, respectively).

Both lipid and fatty acid composition differed significantly among tissue types (Fig 5; PERMANOVA: lipid class, *P* = 0.001; fatty acid composition, *P* < 0.001), spawning area (PERMANOVA: lipid class, *P* = 0.002; fatty acid composition, *P* = 0.001), and year (PERMANOVA: lipid class, *P* < 0.001; fatty acid composition, *P* < 0.001). SIMPER was performed to determine lipids or fatty acids contributing most to the observed differences between two groups. The analysis revealed TAG to be the main differentiating lipid class in muscle and liver tissues (contributing 42.8% and 32.6% to dissimilarity, respectively), and PC to be the main differentiating lipid class in ovary tissues (28.1%) between spawning area. Of fatty acids, 18:1n-9 (oleic acid) and 22:6n-3 (DHA) contributed most to dissimilarity in muscle and liver tissues between spawning grounds (SIMPER; muscle: oleic acid (26%), DHA (21%); and liver: DHA (19%), oleic acid (19%)), whereas differences in fatty acids within ovary tissues were caused by 20:5n-3 (EPA) instead of DHA (SIMPER; ovary: oleic acid, 32%; EPA, 16%).

**Fig 6:**
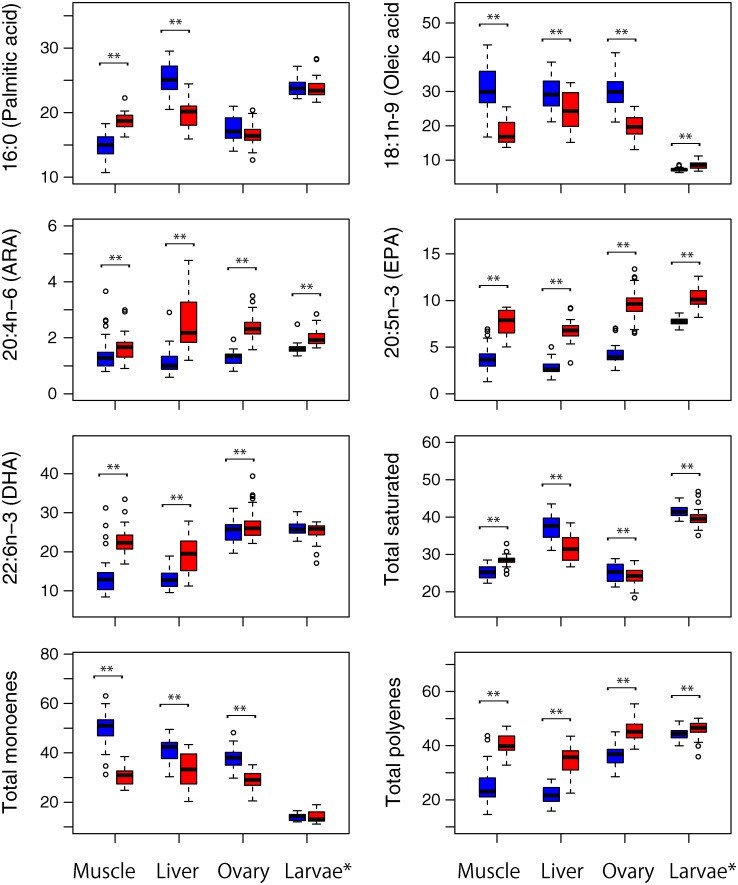
Proportional contribution of individual fatty acids in muscle, liver, ovary tissues, and larvae of Pacific bluefin tuna caught around the Nansei Islands (blue) and in the Sea of Japan (red). Boxes depict the first, median and third quartiles, the lowest and highest datum within 1.5 interquartile range of the first and third quartiles, and outliers (plotted individually). *after Matsumoto et al. [30]. **statistically significant difference (*P* < 0.01, Wilcoxon-Mann Whitney test).

Percentages of the main fatty acids in PBF muscle, liver and ovary tissues, and larvae (after Matsumoto et al. [30]) caught around the Nansei Islands and in the Sea of Japan are shown in Fig 6. Marked and significant differences between spawning grounds are apparent in %DHA, %EPA and %ARA in all maternal tissues, with mean %EPA values in ovary, muscle and liver tissues of PBF caught in Sea of Japan being at least twice those of PBF caught around the Nansei Islands (2.2, 2.0 and 2.3 times, respectively). Although the %DHA in larvae did not differ significantly between spawning grounds, all PBF maternal tissues and larval muscle from the Sea of Japan had higher %EPA, %20:4n-6 (ARA), and total polyenes than tissues from PBF caught around the Nansei Islands. Additionally, large and similar proportional contributions of DHA and total polyenes occurred in ovary tissues and larvae of fishes caught in both spawning grounds, around the Nansei Islands (mean ranges: DHA 24.8%–25.8%, polyenes 36.1%–44.4%) and Sea of Japan (mean ranges: DHA 25.1%–27.4%, polyenes 46.0%–46.2%). However, lower levels of DHA and polyenes occurred in muscle and liver tissues of PBF caught around the Nansei Islands (mean ranges: DHA 12.8%–13.9%, polyenes 21.8%–25.0%).

## Discussion

### Lipid allocation during spawning season

Developing oocytes can obtain lipids from three possible sources: 1) exogenous, supplied through the maternal diet, 2) endogenous, mobilised from adipose fat, and 3) those synthesised de novo in the ovarian follicle from small organic precursors or fatty acids mobilised and transported from other tissues [13, 39]. During oocyte growth, vitellogenin (VTG) is incorporated by the oocyte and processed into yolk proteins [39]. Lipids are delivered to the oocyte by VTG and a variety of plasma lipoproteins, especially very low density lipoprotein (VLDL) [13]. VTG lipids are primarily phospholipid rich in n-3 PUFA, whereas VLDL is rich in TAGs. These lipoproteins are synthesised by the liver, which in the majority of teleost fish then transports most yolk proteins to the developing oocyte [12, 39]. Variation in TL levels in ovary tissues of female PBF in different oocyte developmental stages (Fig 2) is comparable to that reported for other tunas [21, 40–42], being high in Ly and Mn (developing or spawning capable phase), and low in Pn (resting or regressing phase) and Hy (spawning capable phase). Although we did not statistically test for variation in lipid classes, variation in TL might indicate preferential accumulation of certain lipids from the reserves of other tissues during vitellogenesis. The decline in TL during the Hy oocyte developmental stage probably results from an increase in water content due to oocyte hydration, and the process of protein uptake in oocytes, which can stop lipid accumulation during germinal vesicle breakdown [21].

Seasonal patterns in variation of lipid and gonadal indices—the decrease in TL in all tissues and increase in GSI (Fig 3)—indicates the use of lipids for gonad development during the spawning season. The PBF spawning-season duration is estimated to be three months around the Nansei Islands [26] and two months in the Sea of Japan [29]. Although the length of time an individual PBF resides within a spawning ground is unknown, full lifecycle bioenergetic models estimate this to be between 18 and 40.5 days [43]. For the eastern population of Atlantic bluefin tuna (ABT, *T*. *thynnus*), archival tag data has revealed individual fishes reside within spawning grounds for 19–31 days [44]. Therefore it is reasonable to assume that the seasonal change in lipids partly reflect those of variations in individual fishes during spawning season.

In both spawning grounds, the increase in GSI during the early spawning season coincided with a decrease in TL, as did the subsequent levelling of both GSI and TL later in the spawning season (Fig 3). Levels of TAG in liver tissues of fishes from both the Nansei Islands and Sea of Japan, and white muscle in fishes from the Sea of Japan, became highly depleted during the spawning season, while there was little change in TAG levels within ovary tissues (Fig 4). In teleosts, TAG is a major neutral lipid in egg oil globules, and it accumulates within oocytes via the liver before and during the spawning season [12, 13]. Given the reduction in TL during the early spawning season coincides with increased GSI, it is possible that TAG depletion is caused by its allocation from liver and white muscle tissues to oocytes, to ensure appropriate endogenous energy resources are available to offspring. Similar TAG allocations have been reported for other tuna species (yellowfin tuna *T*. *albacares* [21], and albacore *T*. *alalunga* [40]).

PBF in the Sea of Japan might invest more energy into spawning than those from around the Nansei Islands. It is also possible that the total hepatic lipid storage of female PBF from around the Nansei Islands is sufficient to cover their reproductive energetic demands. During the spawning season, the rate of depletion of TAG in PBF white muscle tissue of fishes from the Sea of Japan was higher than it was from fishes around the Nansei Islands (Fig 4). As the TL in PBF liver tissues was greater around the Nansei Islands than it was in the Sea of Japan, especially late in the spawning season (Fig 3), depletion of TAG from white muscle tissues of Sea of Japan fishes may have been required to provide sufficient energy reserves for developing oocytes (TAG reserves in maternal liver tissues would first be allocated, followed by those in white muscle tissues).

The GSI of PBF in the Sea of Japan is significantly higher than it is for PBF from the Nansei Islands (Table 2; Fig 3). A comparison of spawning frequency and relative batch fecundity (RBF) among PBF spawning grounds [45] revealed both to be much higher in the Sea of Japan (mean spawning frequency 0.91, mean RBF 109.8 eggs/g) than around the Nansei Islands (mean spawning frequency 0.3, mean RBF 56.4 eggs/g), although spawning frequencies might be affected by differences in sampling gear. The greater fecundity of younger PBF in the Sea of Japan would require more energy for reproduction. In addition to liver and white muscle, mesenteric perigonadal fat is another source of lipid deposits for gonadal development for ABT [42]. Unfortunately, we did not record total liver weight or collect mesenteric perigonadal fat samples, but we do recommend collection of both for any future study [19, 20].

Our results reveal the proportion of TAG in ovary tissues to remain relatively constant (approximately 25%) throughout the spawning season (Fig 4), but for TL to gradually decrease in ovary tissues in both spawning grounds (Fig 3). Water temperature during the spawning season at both spawning grounds tended to increase from April to July around the Nansei Islands, and to August in the Sea of Japan [8]. A strong inverse relationship between egg diameter and water temperature is known for cultured [46] and wild PBF eggs [10]. Within an optimal temperature range, oocytes would develop faster in warmer waters, and the PBF spawning interval would decrease [10]. This suggests that female PBF would frequently spawn small eggs late in the spawning season due to increased temperature, resulting in decreased TL within ovary tissues, because the time available for lipid allocation had been reduced. Considering growth-dependent mortality in larval PBF, a late spawning season, high spawning frequency might be advantageous, despite individual eggs having reduced TL, because high water temperature promotes larval growth.

### Reproductive strategy of PBF

A capital breeder finances the energetic costs of reproduction using stored capital, while an income breeder finances it with concurrent intake [47, 48]. Which reproductive strategy is followed determines how lipids and fatty acids are allocated from the maternal diet to the larvae during egg provisioning. Therefore, the distribution of lipid classes in somatic and gonad tissues during the reproductive cycle is indicative of the reproductive strategy a species follows [21, 41, 42, 49]. Many species, including yellowfin tuna and albacore, present with a mixture of capital- and income-breeding strategies [21, 40, 47].

During spawning season, a continuous reduction in a condition factor (BW/FL^3^ × constant) of female PBF has been reported around the Nansei Islands [27] and in the Sea of Japan [29]. PBF are iteroparous and thought to produce an average of nine highly variable egg batches a year [43]. Given the TL in liver tissues is greater than in white muscle (Fig 3), and the preferential allocation of TL from liver to ovary tissues (Fig 4), the liver appears to be one of the important lipid storage organs in female PBF. This suggests this species is primarily a capital breeder or mixed capital-income breeder, because a variety of mixed-breeding strategies are possible [47]. Limited information is available on the feeding habits of PBF during spawning season [50]. Therefore, PBF may depend largely on capital to finance reproductive output, and we propose it to be a mixed capital-income breeder, like albacore [40].

### Possibility of maternal effect to the larval survival process

In marine fishes, maternal effects might contribute towards recruitment fluctuation, influencing early larval survival rates. Maternal effects have been previously described using egg size, as larger eggs lead to higher hatching success, higher survival and faster growth [51]. However, the importance of fatty acids to the fatness of offspring, especially n-3 and n-6 long-chain polyunsaturated catty acid (LC-PUFA: essential for normal functioning of larvae), are well established. For example, Perez and Fuiman [18] demonstrated that larval red drum survival, swimming speed, escape response latency and escape response distance, are all significantly correlated with EFA concentration in eggs. Therefore, levels of critical nutrients in eggs might be a valuable metric of maternal investment [14].

We identify differences in lipid and fatty acid composition in different tissues, in different spawning grounds (Fig 5 and S1 Table). Although PC contributed most to any dissimilarity in lipid classes within the ovary, differences in mean values were not large (29.7%–33.7%, Fig 4). In contrast, we demonstrate distinct differences in fatty acids of larvae, especially the increased proportional concentrations of EPA and ARA in the Sea of Japan than around the Nansei Islands (Fig 6). As experimental results have demonstrated that concentrations of certain EFAs in eggs affect larval quality [14, 18, 22], differences in PBF egg quality in different spawning grounds could be a maternal effect that affects offspring survival.

Eggs of PBF spawning in the Sea of Japan are enriched with EPA and ARA relative to eggs from around the Nansei Islands. Many studies have demonstrated that eggs with high LC-PUFA levels, including EPA, promote embryonic survival, hatchability and starvation tolerance [15, 17, 52]. ARA is equally as important, serving as a precursor to eicosanoids associated with egg development [53]. Spawning of PBF in the Sea of Japan occurs at SSTs between 19.3 and 27.7°C, with larvae collected at temperatures exceeding 26°C [24, 29]. Early spawning season larvae hatching in the Sea of Japan might experience lethal variable (low) temperature. In contrast, older PBF spawning in a limited area around Nansei Island maximize the chance of larval survival in nursery grounds, despite less-favourable (warm) water temperatures for adult [54]. Growth trajectory studies have revealed the initial growth of larvae in the Sea of Japan fluctuates greatly and annually, due to abrupt changes in water temperature, with larvae with lower growth rate than those from around the Nansei Islands possibly surviving to recruit in the Sea of Japan [8, 31]. The higher quality of the eggs that we report in the Sea of Japan (they contain more EPA and ARA) will enhance the chances of egg and larval survival in less-favourable environments, such as in low temperatures.

Segregating spawning adults (by age or size) among spawning grounds disperses risks associated with environmental variability, reducing the probability of PBF stock collapse [45]. However, different spawning grounds experience a wide range of water temperatures—one of the most important factors affecting their larval survival [7–9, 31]—variability which also explains changes in recruitment of age-0 PBF [4–6]. Although PBF eggs spawned in the Nansei Islands spawning grounds contain less EFAs, especially EPA, than those in the Sea of Japan (S1 Table), the higher and relatively more stable water temperatures experienced by larvae in this area might offset any adverse effect of egg quality. The difference of %DHA between spawning grounds in ovary tissues was less than that in muscle and liver tissues, but the %DHA did not differ significantly from that found in larvae (Fig 6 and S1 Table). Larger and older PBF from around the Nansei Islands could provide sufficient DHA for egg and larval survival from accumulated energy reserves. In contrast, small and younger adults probably inhabit areas relatively rich in EFAs, the accumulation of which would assist their eggs and larvae to survive in suboptimal temperatures. Fish enhance offspring survival by employing a variety of reproductive strategies [55]. We suggest that PBF use different reproductive strategies depending on variation experienced in their spawning environment.

We provide evidence of a maternal effect in egg quality, and link PBF ovarian and larval fatty acid composition. Ours is, to the best of our knowledge, the first report of a maternal effect in egg quality for a large and highly migratory species. In addition to spatial differences (between spawning grounds), we also report temporal (annual and seasonal) differences in egg quality. TL in 2016 was significantly lower than in other years (Table 2), with lipid and fatty acid compositions also varying in any given year (Fig 5). Although the fatty acid composition of larvae will be affected by their feeding, the accumulation of EFAs in their eggs might ensure normal development, growth and improved stress tolerance during the first several days [15, 17, 52, 53]. Given the highly variable nature of PBF recruitment [3], fluctuation in maternal condition could affect recruitment levels. Further analyses of lipids and fatty acids will likely reveal causes of geographical and annual difference in egg quality, and effects on PBF reproductive potential and recruitment.

## Supporting information

S1 Table Mean, standard deviation (S.D.) and P-values of fatty acids (as % of total fatty acids) in total lipids in ovary, muscle and liver tissues of female Pacific bluefin tuna caught around the Nansei Islands and in the Sea of Japan. Totals include some minor compornents that are not shown.
(XLSX)
